# Coffee Poisoning

**Published:** 1901-01-26

**Authors:** 


### COFFEE POISONING.

Dr. William M. Leszynsky,[1] of New York, holds that although "coffee when well prepared is beyond question one of the ambrosial luxuries of modern life," yet there is danger in the cup unless taken with somewhat more of judgment than is usually expended upon it. While to the majority its taste and action are agreeable, for others it is really a poison—a matter which is all the more important in consequence of the price having undergone a material and progressive decrease during the past eight or nine years, which, among other things, has led to a rapid increase in its consumption. The people of the United States consume, it is said, about one-third of the total coffee production, and this we are told "may account in great measure for the prevailing nervous characteristics of many of our population, their increasing mental activity, and the actual accomplishment of work that might otherwise be impossible." Dr. Love,[2] writing some years ago on the use and abuse of coffee, went so far as to say that "in excess it is even more dangerous than alcohol, for it is not, like the latter, a nutrient, nor is the effect of its excessive use so apparent or unrespectable." The intellectual faculties excited to the greatest degree by the use of coffee are, we are told, the imagination and the memory. It produces an augmentation in the power of attention, a vivacity of thought and conception, increased capacity for mental and physical work, and transitory ambitions often beyond the physical or mental capability of the individual. Hence many dangers. It is not merely that the taking of coffee enables some people to wear themselves out more quickly than they would have done without it. Besides this it may produce directly deleterious effects, among which may be noted a feeling of apprehension and general tremulousness; disturbance of digestion; paroxysmal sneezing and coryza, or itching of the skin; wakefulness, and corresponding exhaustion of the nervous centres, neurasthenia, palpitation and rapid pulse; while in acute coffee poisoning there may be excitability to the extent of delirium. We do not think that coffee is often taken in this country to the extent mentioned by Dr. Leszynsky, but one cannot but observe that many of the symptoms which he describes are similar to those which we commonly associate with excessive tea drinking.

[1] New York Med. Rec., Jan. 12. [2] Jour. Am. Med. Assoc., Feb. 1891.

